# Taxonomy of psychopathology based on a neurochemical framework

**DOI:** 10.1192/j.eurpsy.2023.363

**Published:** 2023-07-19

**Authors:** I. Trofimova

**Affiliations:** Collective Intelligence Lab, Department of Psychiatry and Behavioural Neurosciences, MCMASTER UNIVERSITY, Hamilton, Canada

## Abstract

**Introduction:**

Temperament and mental illness are considered to be variations along the same continuum of imbalance in the neurophysiological regulation of behaviour.

**Objectives:**

This presentation presents the benefits of constructivism approach to psychiatric taxonomies.

**Methods:**

The presentation reviews findings in neurochemistry that link temperament traits in healthy individuals and symptoms of psychiatric disorders to complex relationships between neurotransmitter systems.

**Results:**

Specialization between neurotransmitter systems underlying temperament traits is analyzed here from a functional ecology perspective that considers the structure of adult temperament corresponding to the functional structure of human activities. In contrast to a more popular search for neuroanatomic biomarkers of psychopathology and temperament traits in healthy individuals, this presentation focuses on neurochemistry-based biomarkers. The roles of monoamine neurotransmitters (serotonin, dopamine, noradrenalin), as well as the roles of acetylcholine, neuropeptides and opioid receptor systems in the regulation of specific dynamical properties of behaviour are summarized within the neurochemical Functional Ensemble of Temperament (FET) model (Table 1) (Trofimova & Robbins, Neurosci Biobehav Rev, 2016, 64, 382-402; Trofimova, Neuropsychobiology, 2021, 80(2), 101-133).

**Image 2:**

**Figure:**
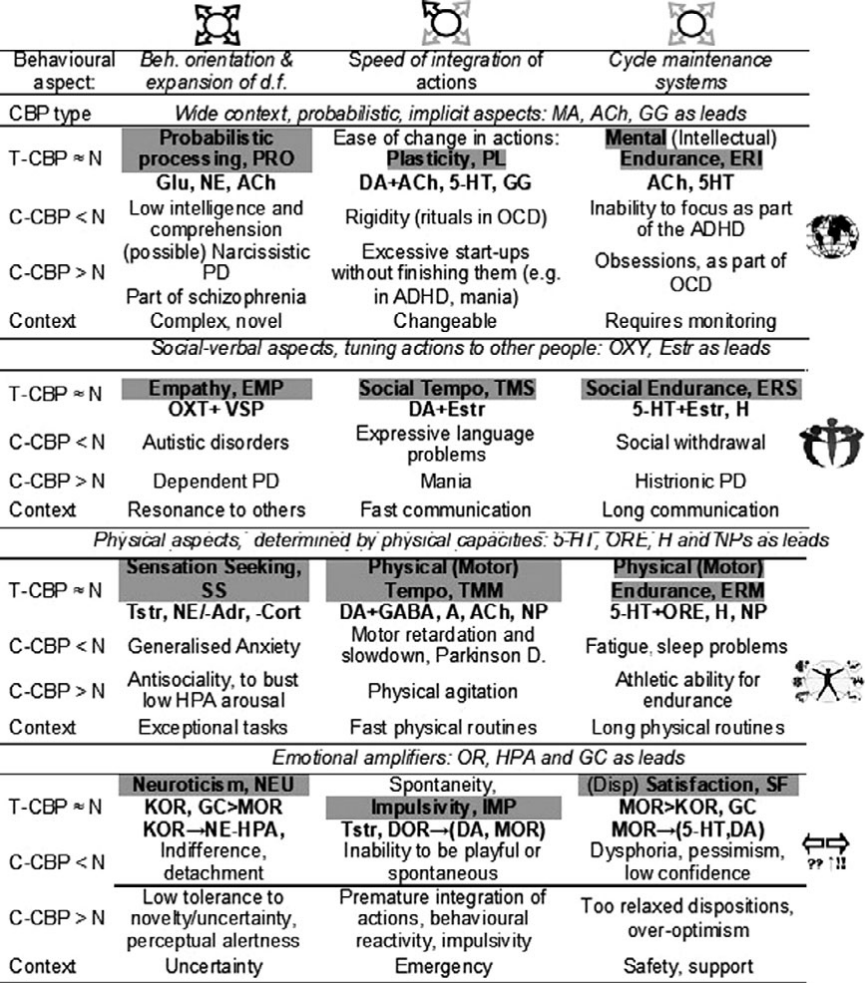

**Conclusions:**

The FET framework allows having a neurochemistry-based structure of a taxonomy that can classify both, healthy bio-psychological traits and symptoms of psychopathology. The presentation will give examples of how the FET framework can be used in psychiatry and clinical psychology.

**Disclosure of Interest:**

None Declared

